# Contrast efficacy of novel phase convertible nanodroplets for safe CEUS imaging

**DOI:** 10.1038/s41598-024-66163-1

**Published:** 2024-07-12

**Authors:** R. Riaz, S. Shafiq, M. Fatima, M. A. Siddique, S. Shah, S. R. Abbas

**Affiliations:** 1 Department of Microbiology and Industrial Biotechnology, Atta ur Rahman School of Applied Biosciences (ASAB), National University of Sciences and Technology (NUST), Islamabad, Pakistan; 2 Present Address: Medical Imaging Technology, FRAHS, Riphah International University, Islamabad, Pakistan; 3 Faculty of Veterinary & Animal Sciences, PMAS UAAR; Maaz Pet Hospital, Rawalpindi, Pakistan; 4 Shifa International Hospital, Islamabad, Pakistan; 5 Biosensors and Therapeutics Lab, School of Interdisciplinary Engineering and Sciences (SINES), National University of Sciences and Technology (NUST), Islamabad, Pakistan

**Keywords:** Nanodroplets, Echocardiography, Ultrasound, Sonovue, Poly glycerol sebacate, Contrast agent, Preclinical research, Nanoparticles

## Abstract

Microbubble contrast agents in ultrasound/echocardiography are used to increase the echogenicity of the target tissues, thereby raising the contrast resolution of the resultant image. Recently, the trend has shifted toward the development of phase-convertible nanodroplets as ultrasound contrast agents due to their promising theragnostic potential by switching capability at the active site. Herein, we fabricated pre-PGS- perfluoropentane phase convertible nanodroplets and checked their in vitro and in vivo enhancement and safety profile. For this, we performed experiments on 20 male Wistar rats and 2 dogs. Biochemical assays of both rats and dogs included complete blood profiles, liver function tests, and renal function tests. For rat vitals, monitoring and histopathological analysis were also performed. Converted nanodroplets showed excellent contrast enhancement, better than Sonovue upon in vitro testing, with an enhancement time of up to 14 min. In vivo, experiments showed comparable opacification of the ventricles of both rats and dogs. All biochemical assays remained within the normal range during the study period. The histopathological analysis did not show any signs of drug-induced toxicity, showing the safety of these nanodroplets. Pre-PGS-PFP nanodroplets hold great potential for use in echocardiography and abdominal imaging in both human and veterinary applications after clinical trials.

## Introduction

As a safe, non-invasive, non-ionizing, real-time, fast and cost-effective mode of imaging soft tissues, ultrasound/echocardiography is the prime imaging modality for diagnosing soft tissue disorders. However, when compared to magnetic resonance imaging (MRI) and computed tomography (CT), USG has lower contrast resolution^1^. In ultrasound, contrast resolution is dependent upon acoustic impedance between objects^2^. Microbubble contrast agents for ultrasound/echocardiography are used to increase the impedance mismatch between tissues, raising the echogenicity of the target tissues and thereby improving the contrast resolution of the resultant image. Since the first report of contrast enhancement by Gramiak & Shah in 1969 via saline bubbles, work has focused on increasing the stability and echogenic response of microbubbles^3,4^

Currently available microbubble contrast agents are micro sized core–shell particles. The core of microbubbles (MBs) consists of any gas with low solubility, while the shell is made of stabilizing material, mostly lipids, polymers, surfactants and proteins. Their echogenic properties lie in their dynamic response in an acoustic field. Upon application of ultrasound, they undergo rapid volumetric oscillations, resulting in more reflection of sound than rigid particles of the same size^5^. All commercially approved contrast agents have a core of perfluorocarbon and a shell made up of lipids. Among perfluorocarbons, the trend has been moved towards using phase convertible gases due to their switchable mode. As these liquid PFCs change into a gaseous state depending upon the boiling point of PFC, insonation frequency and intensity, their size increases up to 10 times inside the body, causing drift towards developing nanodroplets. The use of these nanodroplets provides an added advantage of extravascular imaging. On-site activation of phase convertible nanodroplets (PCNDs) by controlling ADV has opened new avenues for theragnostics and targeted imaging^6^. The nanodroplets can be converted into microbubbles at the targeted site by providing the required insonation frequency or temperature. The microbubbles also have the ability to recondense immediately to nanodroplets after ADV^7^. For diagnostic purposes employing low frequencies, perflouorpentane as a phase convertible agent is a good choice because of its low ADV^8^.

For shell material, lipid-based microbubbles are preferred due to their flexible nature, which provides better oscillations in the diagnostic acoustic field (3–10 MHz), thereby providing increased echogenicity, but they are less stable. For theragnostic applications, the trend has been moving towards developing microbubbles with polymers as shell materials due to their increased stability and easy functionalization. However, due to the high modulus of polymers, they usually oscillate at higher frequencies and cannot be employed in the diagnostic frequency range (less than 10 MHz)^9^.

The echogenicity of polymeric microbubbles in diagnostic imaging frequency can be enhanced by making the shell of microbubbles with polymers having a low elastic modulus, i.e., elastomers, so that they may oscillate at lower frequencies. Various elastomers have been employed in the field of regenerative medicine and drug delivery; however, their use in the field of acoustics is limited^10^. To date, the only elastomer reported as a shell for microbubble contrast agents was polyvinyl alcohol (PVA). A fivefold lower concentration of PVA microbubbles showed a similar response to that of Sonovue microbubbles^11^. However, the bubbles showed an increase in stiffness when stored, and the microbubble resonance frequency changed from 11.5 to 19.7 MHz on the 75th day^12^.

Polyglycerol sebacate (PGS) is a biocompatible and biodegradable elastomer prepared via a two-step melt condensation technique and is extensively applied in tissue engineering applications^13^. An increase in interest in this material is due to its tunable mechanical and degradation properties by controlling its degree of esterification by synthesis conditions^14^. Attractive aspects of polyglycerol sebacate (PGS) for use as an ultrasound contrast agent lie in its excellent viscoelastic properties, i.e., low elastic modulus. This gives the acoustic properties of lipid microbubbles while maintaining the stable response of polymeric microbubbles. Since PGS is insoluble, we utilized pre-PGS as the shell material for PCNDs. In the first step of theoretical modelling, PGS MBs gave much better oscillations in the simulated acoustic field with a higher scattering cross-sectional area than standard Sonovue microbubbles^15^.

PCNDs are now increasingly employed as theragnostic and multimodal contrast agents^16,17^. Pre-PGS, due to its free hydroxyl groups, can be modified with target moieties or loaded with nanoparticles, making it an excellent platform for theragnostic or multimodal applications. In one of our works, PEG-coated SPION-loaded pre-PGS shell-based PCNDs showed potential for use as a T2-MR and ultrasound contrast agent^18^. In terms of improved stability and surface modification, pre-PGS-based PCNDs hold a promising future. However, to be injected inside the body, they need to be completely safe. Intravenous injection of PCNDs raises potential safety concerns in diagnostic ultrasound imaging. Effects from contrast agents range from minor self-resolving effects, including altered taste, headache, nausea, and sensation of heat, to major anaphylactic reactions, embolism and death^19^. As converted MBs resonate in an acoustic field, they can set up a streaming pattern in blood plasma that can alter membrane permeability and can alter the transport of molecules and ions across the membranes. Large amplitude oscillations could lead to heating, i.e., thermal effects, and can generate pressure locally, which may lead to free radical formation. Inertial cavitation at high MI can lead to shear stress in surrounding structures, resulting in damage to the cells and microbleeds^20^. Therefore, in vivo studies are of crucial importance to bring novel PCNDs forward to clinical use.

The current paper is focused on the in vivo contrast efficacy and safety establishment of pre-PGS-based PCNDs since in vivo toxicity cannot be predicted based only on bulk polymer properties or in vitro experiments. The current study was performed in rats with saline as a positive control to test contrast efficacy and any systemic or organ-specific toxicity. The results were then verified on a large animal, i.e., dog with standard Sonovue microbubbles, as a positive control for contrast enhancement.

## Results and discussion

### Physicochemical characterization of PCNDs

**Figure 1:**
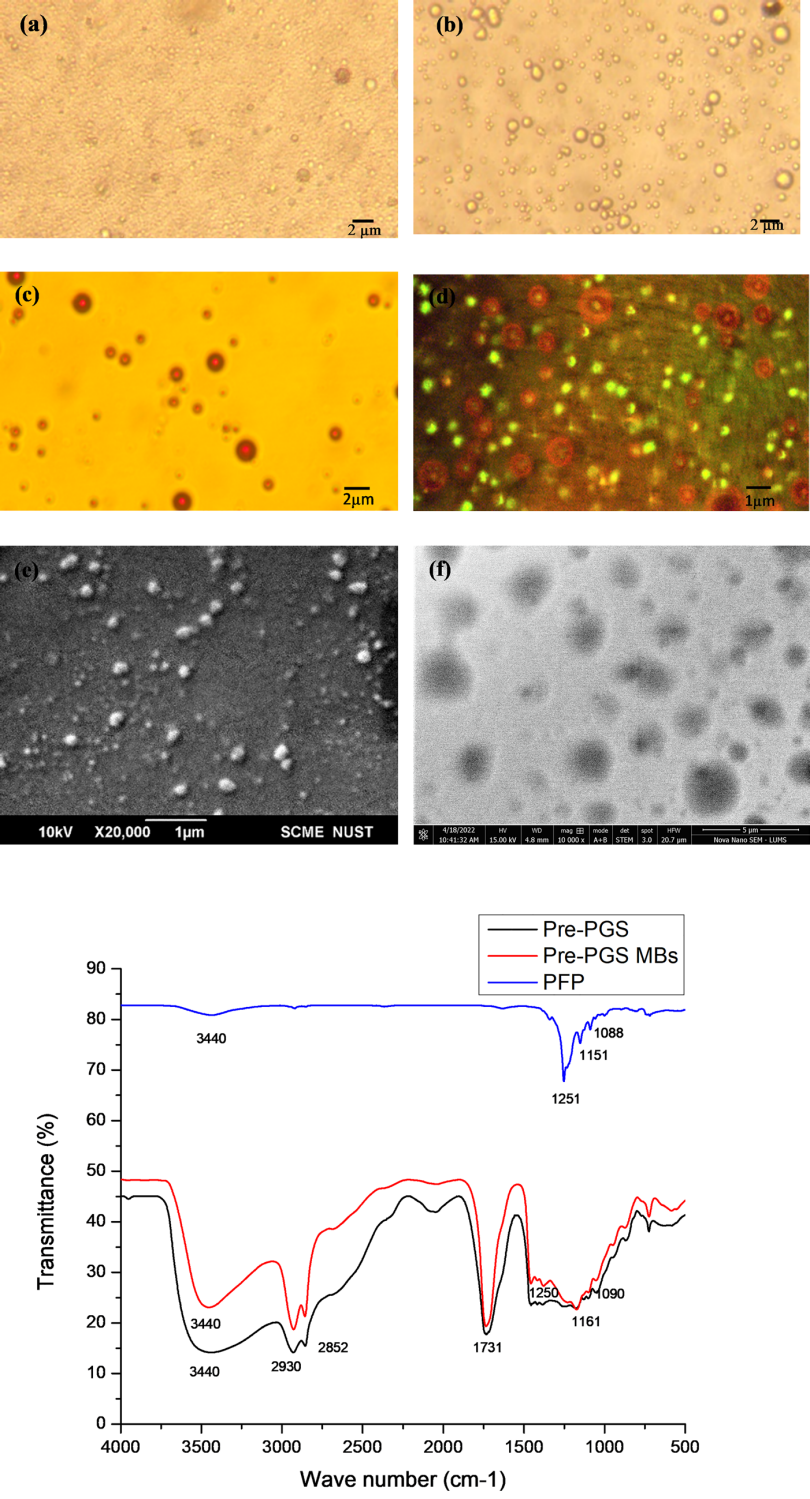
Physicochemical characterization of pre-PGS PCNDs. (**a**) Optical microscopy images of nonconverted nanodroplets. (**b**) Phase-converted microbubbles. (**c**) Fluorescently converted MBs under UV light. (**d**) Confocal microscopy. (**e**) SEM images of nanodroplets and (**f**) FE-SEM micrographs of pre-PGS microbubbles. (**g**) depicts the FTIR analysis of pre-PGS PCNDs with an ester peak at 1731 cm-1.

Optical microscopy of nanodroplets showed phase conversion properties. Heated droplets converted into microbubbles with a size increase of approximately 5–10 times, as shown in Fig. 1a,b. Fluorescence microscopy of phase-converted nanodroplets, i.e., microbubbles, showed core–shell morphology. Nonconverted nanodroplets were invisible under light microscopy due to their size being in the nanoscale range. Images of phase-converted fluorescent microbubbles are shown in the first row of Fig. 1c,d. In SEM (Fig. 1e), nonconverted nanodroplets appeared smooth and spherical in shape with an average size of 189 ± 44 nm, while in FE-SEM, phase-converted nanodroplets/microbubbles appeared spherical in shape with a maximum size of up to two microns (Fig. 1f). Zeta size analysis showed an average diameter of 338.3 nm. Three peaks were observed in the zeta size measurement: peak 1 in the nanometer range (210 ± 66.9 nm with 67.1% intensity) is attributed to non-phase converted nanodroplets, while two peaks in the micrometer range, i.e., at 1061 ± 288.1 nm and 5359 ± 341.6 nm with intensities of 27.3% and 5.1%, respectively, are due to phase converted microbubbles. Zeta analysis of liquid perfluorocarbons (PFCs) always shows multiple peaks near the boiling point, as phase conversion is relatively dependent on many parameters, including the boiling point. Additionally, the literature shows that a hundred percent phase conversion can never be achieved as microbubbles recondense immediately^21^. Since zeta analysis was performed at room temperature, i.e., 25 ℃, which is near the boiling point of PFP due to the lack of a temperature-controlled instrument, similar findings were observed in the current study. This small size compared to Sonovue MBs makes them more permeable to the capillary bed and fewer chances for embolization. The zeta potential of the synthesized nanodroplets was found to be −19.3 mV, indicating the presence of hydroxyl groups in the prepolymer.

FTIR analysis of synthesized nanodroplets showed an ester peak of the polymer at 1731 cm^−1^. The nanodroplets showed PFP stretching between 1000 and 4000 cm^−1^, corresponding with the FTIR spectrum of PFP shown in black (Fig. 1g), indicating encapsulation of PFP in pre-PGS nanodroplets.

### In vitro echocardiographic imaging

**Figure 2:**
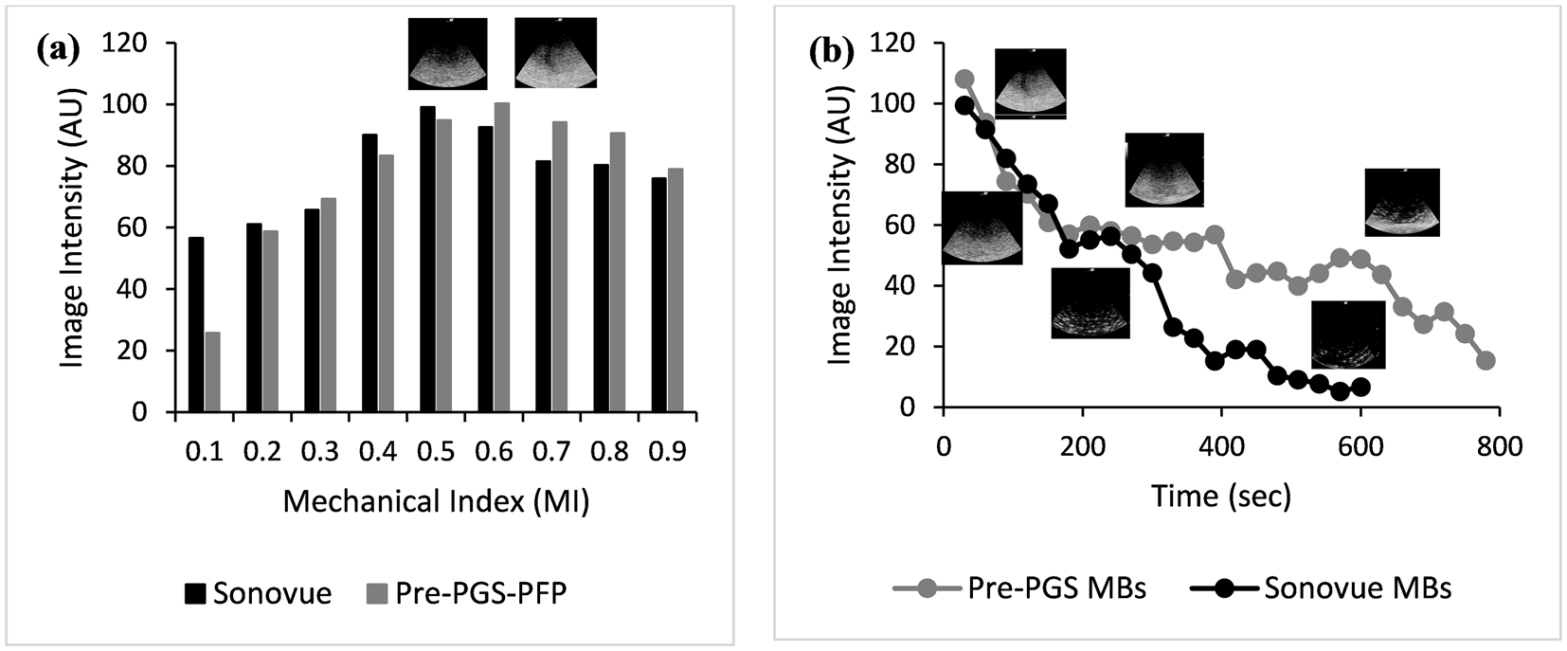
In vitro enhancement analysis. (**a**) shows pre-PGS MBs intensity to be at par with Sonovue MBs, (**b**) shows time intensity curves of pre-PGS-PFP MBs versus Sonovue MBs with pre-PGS-PFP MBs showing almost double enhancement time than Sonovue MBs.

Phase-converted pre-PGS microbubbles (MBs) showed at par enhancement to Sonovue MBs on in vitro image evaluation with an increase in intensity up to 100 times. Above 0.5 MI, it showed slightly higher image intensity than Sonovue MBs. The duration of enhancement by pre-PGS MBs was almost double that of Sonovue MBs. Sonovue MBs showed an enhancement time of 4 to 8 min, as reported in the fact sheet^22^, while pre-PGS-PFP microbubbles showed an enhancement time of 10 to 14 min (Fig. 2).

### In vivo echocardiography in rats

A total of 20 male Wistar rats were included in the study and were broadly divided into three groups: a control group, a saline group and a contrast group. The contrast group was further divided into a 1st-day dissection group and a 10th-day dissection group for safety evaluation. A detailed division is explained in the methodology section.

**Figure 3:**
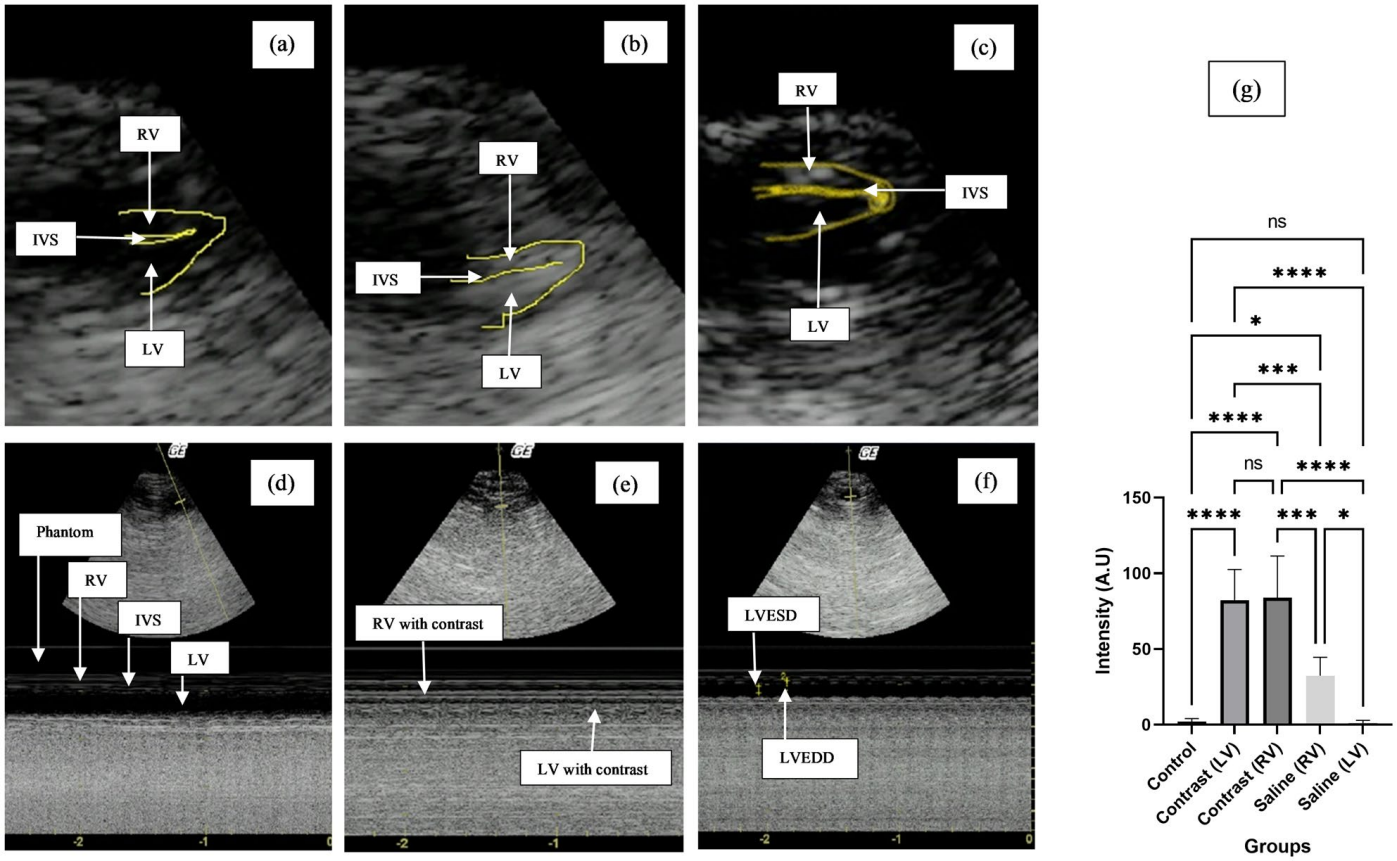
Echocardiography images; (**a**) shows B-mode pre-contrast echocardiography, (**b**) shows postcontrast B-mode image, (**c**) shows post-saline B-mode image, (**d**) is pre-contrast M-mode image, (**e**) is postcontrast M-mode image and (**f**) is post-saline M-mode image, (**g**) image depicting ANOVA on pre- and post-contrast images showing significant enhancement by pre-PGS MBs in comparison to saline and pre-contrast (control) images.

To check the in vivo left ventricle opacification potential, pre- and postcontrast images were taken. Postcontrast images were also compared with agitated saline-enhanced ventricles. Agitated saline echocardiography has been in practice since 1968 due to its cost-effectiveness; however, it only opacifies the right ventricle due to its inability to cross the pulmonary vasculature^23^. In this in vivo study, echocardiography images of pre-PGS PFP MBs showed almost 80 times enhancement of both ventricles, while that of saline MBs showed 30 times enhancement of only the right ventricle compared to the pre-contrast image. Figure 3 shows the pre-contrast and postcontrast B-mode and M-mode echocardiography images of rats and ANOVA on contrast enhancement.

Significant contrast enhancement was observed between the control and saline-administered right ventricles (P value = 0.03) and the contrast and saline groups in both the right and left ventricles (P value = 0.0004). No change (P value = 0.99) was observed between the control and left ventricle image intensity upon saline administration.

### Safety establishment of pre-PGS PFP PCNDs in rats

According to the European Medical Agency, safety analysis of drugs in an animal model is necessary before the clinical trial journey. These safety assays must be able to assess any potential deleterious effect on vital systems of the body, i.e., cardiac, pulmonary, renal, respiratory, nervous, and hepatic systems. Therefore, they recommend both biochemical profiling and histopathological analysis to study organ-specific toxicity^24^.

#### Vitals monitoring

**Figure 4:**
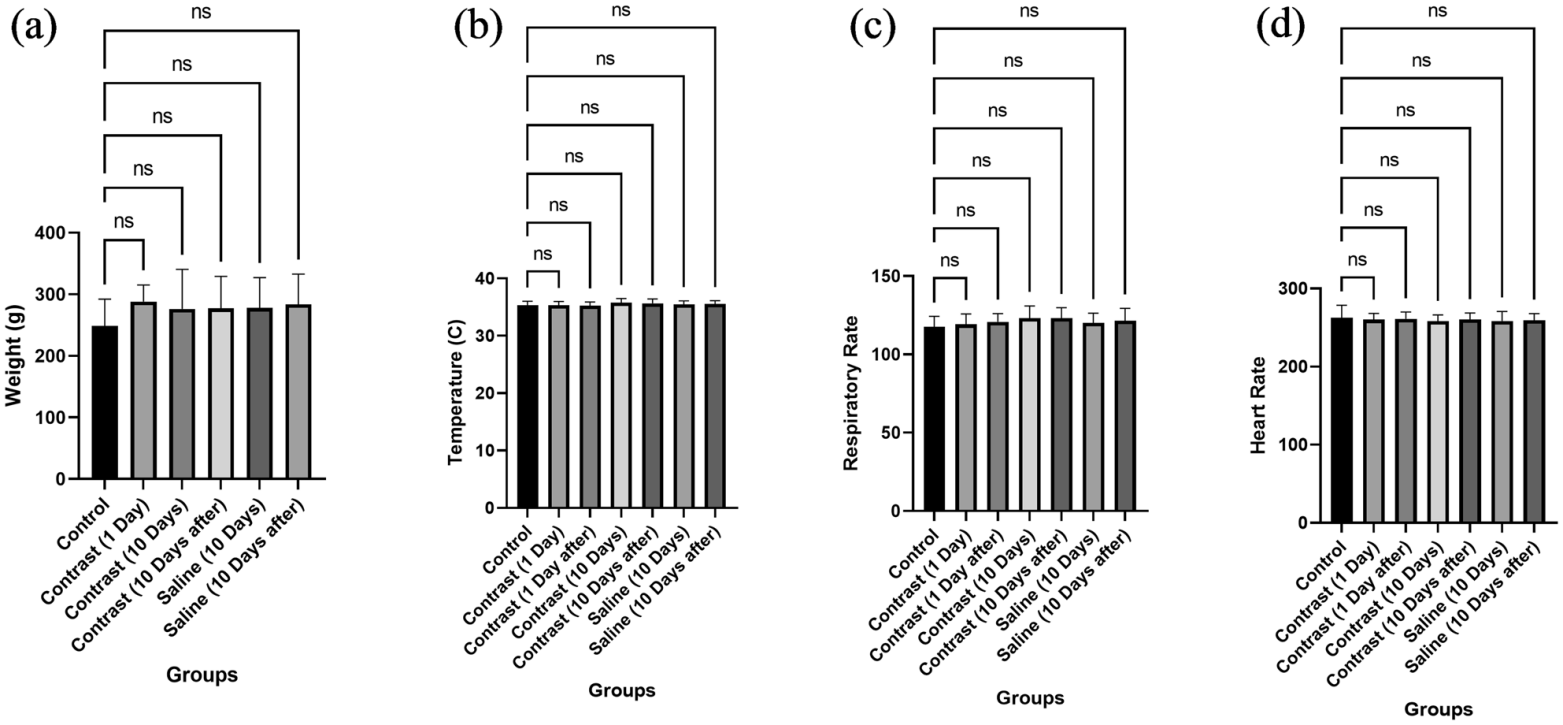
All groups showed normal vitals. The P value was considered significant at P < 0.05. ns represents nonsignificant results.

The severe acute response towards contrast agents includes IgE-mediated anaphylaxis and complement-activated pseudo allergic reactions (CARPA). Both may result in severe respiratory distress, deleterious CNS effects, cardio-pulmonary arrest, and arrhythmias. The initial signs and symptoms of these reactions include a high pulse rate, difficult breathing, mucosal secretions, skin rashes, skin swelling, vomiting, and diarrhoea, leading to lethal complications within minutes of contrast administration if left untreated^25–27^. Therefore, we monitored all vital parameters before and after contrast injection. The results showed that all rats remained completely healthy during the study period. No signs of allergic response, i.e., nasal, ear, or ocular secretion, change in texture, or colour of skin, were observed during the whole study period. No change in sleep or dietary pattern was noted. Vital monitoring, i.e., temperature, respiratory rate, heart rate, and weight of animals, did not show any significant variation between the groups, as depicted in Fig. 4a–d.

#### Biochemical profile of rats

**Figure 5:**
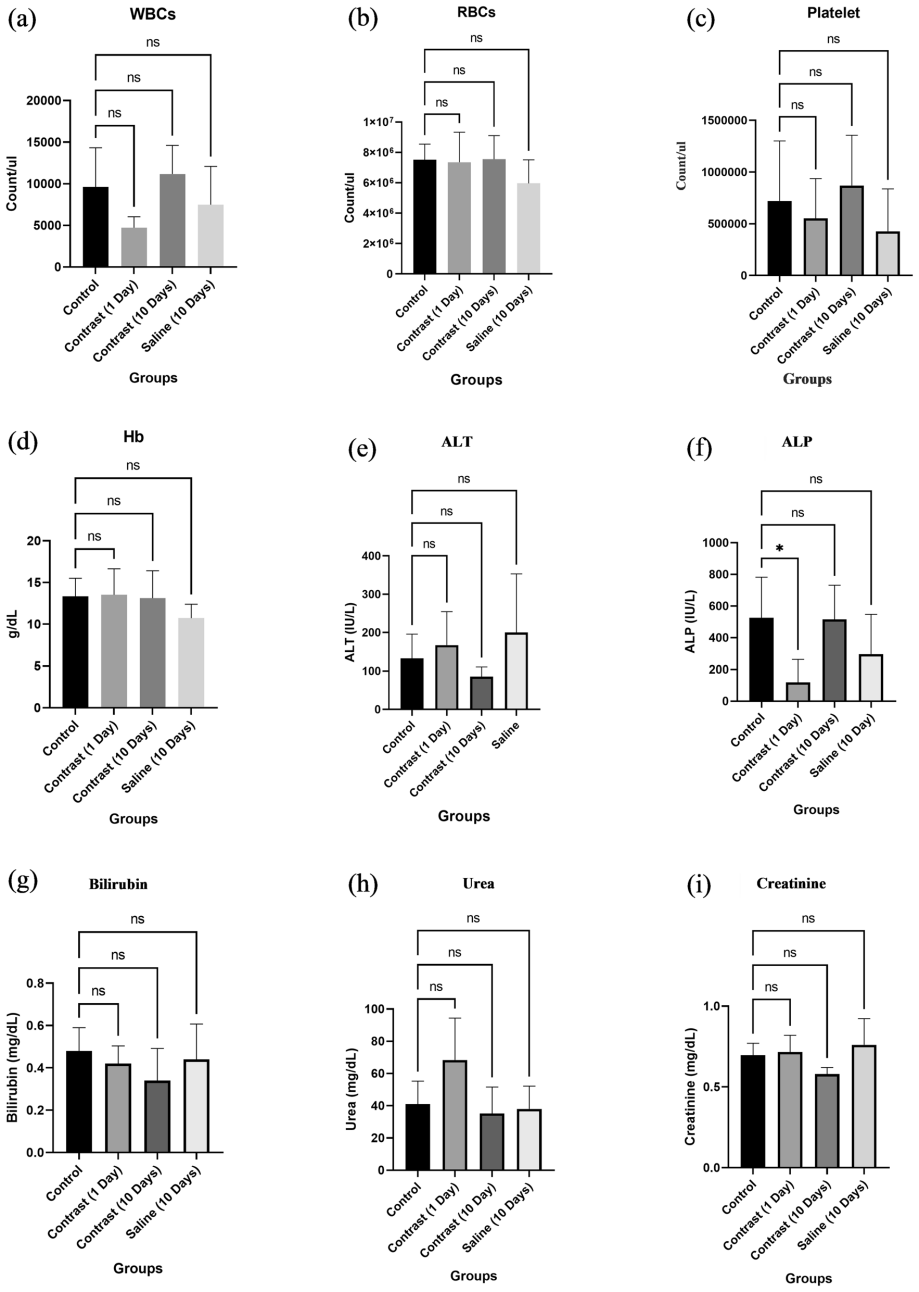
ANOVA of biochemical assays of rats. The P value was considered significant at P < 0.05. NS represents nonsignificant results, while significant results are represented by *.

Complete blood count (CBC), liver function tests and renal function tests of all the study animals were performed to evaluate any potential adverse effects of pre-PGS PCNDs. CBC was performed because it is the simplest, cheapest, and most easily available test for predicting infections, coagulopathies, acute haemorrhagic states, immunodeficiencies and acute and chronic inflammatory responses^28^. For acute and chronic inflammatory responses, WBCs are usually increased, as they are the key players against pathogens and injury^29^. Within leucocytosis, neutrophilia occurs as a result of acute bacterial infections, eosinophilia, and basophilia as a response to allergic reactions, monocytosis due to viral infections, and lymphocytosis in chronic responses^30^. No leucocytosis was observed in our findings. Other important indicators in CBC include red cell count and haemoglobin levels, which provide an idea regarding haemolysis and anaemic conditions, while platelet count tells us about coagulopathies and other inflammatory conditions^29^. No significant variation was found between the control group and other groups for hematological parameters (Fig. 5a–d).

Liver function tests were performed because they play a key role in detecting drug-induced liver injuries. Fivefold-fold elevation of serum alanine aminotransferases (ALT) and twofold-fold elevation of alkaline phosphatase and bilirubin indicate drug-induced toxicity^31^. Since normal ranges vary from lab to lab, we compared all test groups with the control group. No significant variation was found between the control group and test group for ALT and bilirubin; however, ALP values were significantly decreased in the postcontrast 1 day group (Fig. 5e–g).

For non-invasive detection of nephrotoxicity, renal function tests were performed, which included serum urea and creatinine levels. Serum urea is an indicator of global kidney function, while creatinine levels are indirectly linked with glomerular filtration rate^32^. All rat groups showed normal biomarkers, indicating nephrotoxic effects (Fig. 5h,i).

#### Histopathological analysis of vital organs

Histology is considered the gold standard for diagnosing organ-specific toxicity. Therefore, we performed histopathological analysis of five vital organs, i.e., the liver, kidney, heart, brain, and lungs. Since nanodroplets are also taken up by macrophages, we also performed histology of the spleen. Before histopathological analysis, every organ was weighed and compared. No significant difference was found between the organ weights of the different groups (Supplementary Table 1).

**Figure 6:**
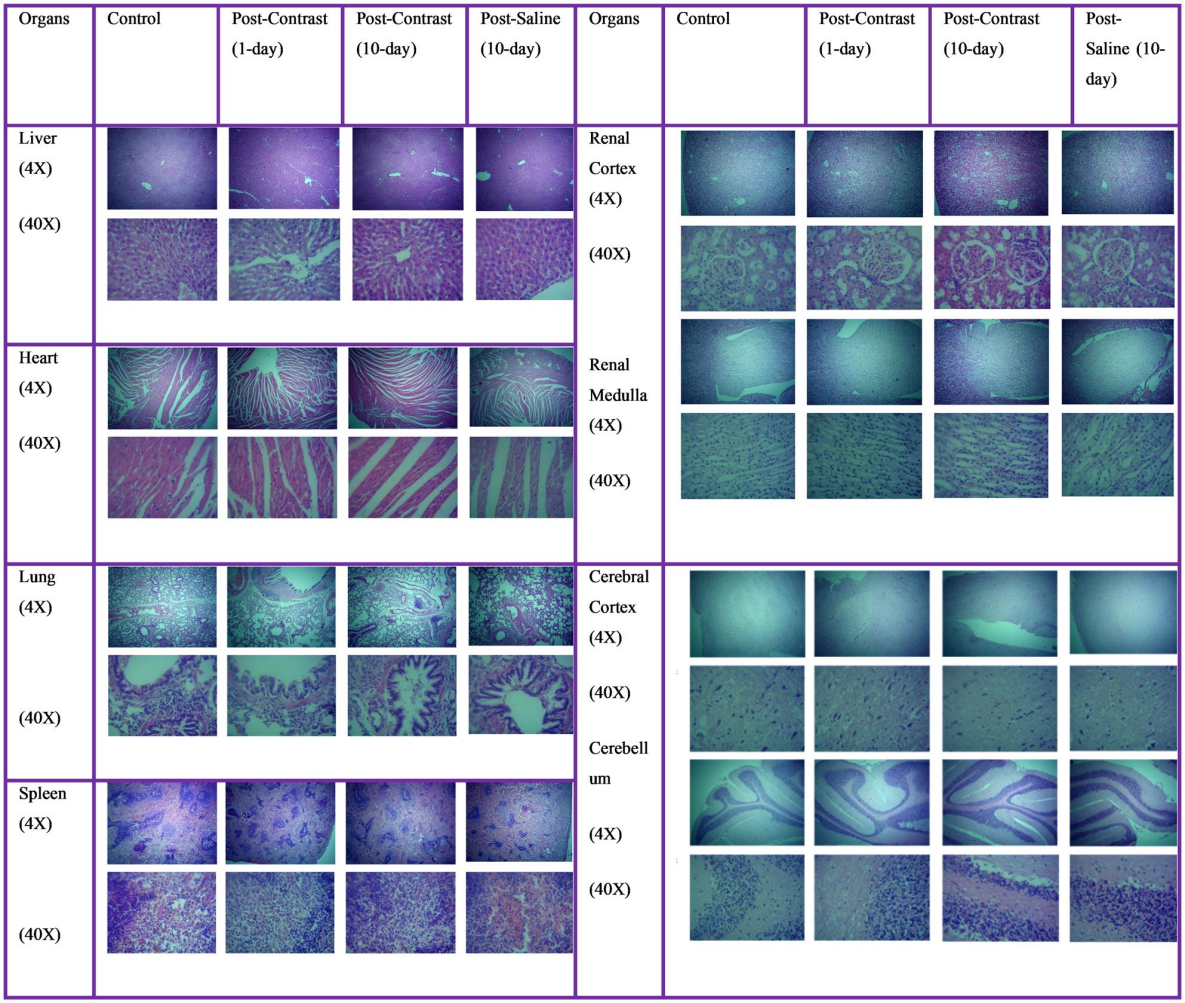
Histological sections of liver, heart, kidney, lungs, spleen, and brain showing normal anatomy.

Liver histology was performed to check for any potential drug-induced liver injury (DILI). DILI refers to a diverse set of responses that arises as a consequence of the administration of pharmacological agents. It can lead to fulminant hepatic failure, resulting in hepatic encephalopathy and coagulopathy^33,34^. Thus, it is recommended to assess drug-induced damage at the tissue level in preclinical trials. The normal liver shows hexagonal lobular anatomy with a central vein located in the centre of the hexagon in the histology studies. Hepatocytes appear radiating from the central vein in a cord-like fashion. Drug-induced liver injury usually manifests as acute hepatitis, chronic hepatitis, acute cholestasis, and chronic cholestasis^35^. All visualized liver sections of both the control and contrast groups showed normal liver architecture. No areas of coagulative necrosis or lymphoid aggregates were observed. No signs of acute cholestasis, i.e., loss of normal parenchymal sinusoidal architecture lobular disarray, hepatocyte dropout, or rosette formation, were observed. Biliary canals did not show any signs of cholestasis (Fig. 6).

Nephrotoxicity is one of the major concerns encountered during preclinical trials, as the kidney is involved in the removal of toxins, drugs, and other excretory materials. It mostly manifests as interstitial nephritis, tubular nephritis, and glomerular disease^36^. Visualized histological sections of renal tissue showed normal cortical and medullary regions with cortical regions showing normal proximal and distal convoluted tubules, normal glomeruli with intact Bowman’s capsules, and stripped medullary regions with ducts. No signs of nephritis, i.e., infiltration of leukocytes, loss of brush border, tubular dilatation, prominent nuclei, and cytoplasmic vacuolization, were noted. Similarly, the glomerulus did not show any signs of glomerulopathies, i.e., retraction of the glomerular tuft, basement membrane vacuolization, or podocyte proliferation (Fig. 6).

Cardiotoxicity is another lethal complication encountered as a result of drug administration. In histology, drug-induced injury to cardiac tissue appears as ischemic changes with necrosis of cardiomyocytes, subendocardial necrosis, or eosinophilic degeneration of muscle fascicles with or without hemorrhage. Old ischemic insult can appear as fibrous scarring with aggregates of macrophages^37^. No such change was noted in our study sections. All sections showed normal cardiac tissue appearance with three distinct histological layers. The muscles showed a typical striated appearance with beautifully arranged mononucleated cardiomyocytes (Fig. 6).

Neurotoxicity as a result of drug administration can lead to severe morbidities, including dementia, convulsions, coma, paralysis, and loss of major functions, such as loss of speech, vision, or hearing^38^. Histological sections of the brain showed a normal cerebral cortex and cerebellum without any zones of ischemia, hemorrhage, neuronal loss, or inflammation. All three cerebellar layers were visualized with the granular cell layer in dark blue, the molecular layer in pink with dispersed Purkinje cells, and the white matter underlying these folia (Fig. 6).

Pulmonary toxicity by the drug can result in both acute and chronic pulmonary disease, i.e., it may cause diffuse alveolar damage, nonspecific interstitial pneumonia, bronchiolitis obliterans organizing pneumonia, eosinophilic pneumonia, nonspecific interstitial pneumonia, veno-occlusive disease, and haemorrhage. Normal pulmonary tissue in H & E staining appears largely as hollow space with thin-walled alveoli. The pseudostratified epithelium of bronchi appears blue, while the smooth muscle layer appears pink^39,40^. Our slides also showed a similar appearance without any signs of pneumonia, i.e., no patchy expansion of interstitium, honeycombing, thickening of alveolar septa, or infiltration of leukocytes was seen (Fig. 6).

Since the majority of nanoparticles are taken up by the spleen and our nanodroplets were on the order of nanometers when administered and converted into microbubbles to achieve the ADV threshold, we performed histology of the spleen to check for any potential injury to the spleen. Microscopically, splenic injury appears as a change in the normal distribution of white and red pulp. Some chemicals may result in fibrosis of the parenchyma, lipidosis, or haemorrhage^41,42^. No such changes were observed in our study groups, with all sections revealing normal parenchymal architecture with trabeculae having arteries and veins. The dense sheath of lymphocytes could be seen encircling arterioles, making white pulp blue and the bulk of the spleen pinkish-red, constituting red pulp (Fig. 6). Histology of all six major organs confirms the safety of pre-PGS MBs. Based on these safety analyses, we took one step forward and established our enhancement and safety in dogs.

### Verification in dogs

#### Contrast enhancement of pre-PGS-PFP in comparison with Sonovue microbubbles in dog

**Figure 7:**
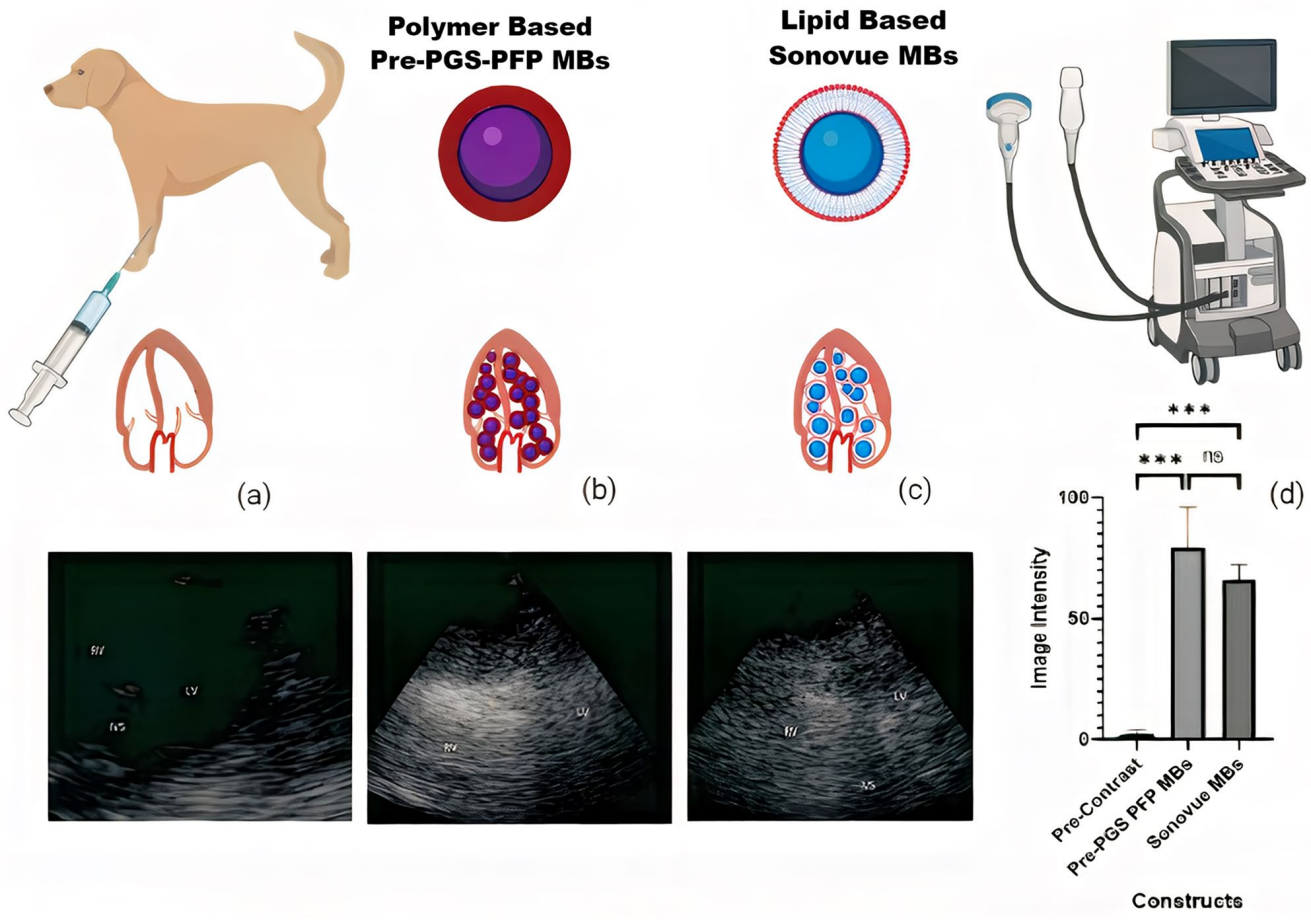
Pre-contrast echocardiography image (**a**). IV administration of polymer-based pre-PGS-PFP MBs showed higher enhancement (**b**) than lipid-based Sonovue MBs (**c**). ANOVA of echocardiograms showing significant contrast enhancement by both pre-PGS-PFP.

The contrast enhancement potential of pre-PGS MBs was further verified by injection in dogs and comparison with dogs receiving standard commercial Sonovue contrast agent. Both Sonovue and pre-PGS-PFP MBs showed comparable contrast enhancement in echocardiography, with pre-PGS-PFP MBs showing slightly higher enhancement than Sonovue microbubbles (Fig. 7).

Pre-PGS-PFP MBs gave a longer imaging window. This provided us with an opportunity to image the liver and kidney of the dog after five to six minutes of echocardiography. The liver retained contrast and showed slow washout. However, when the kidney was observed after the liver, very little contrast was present in the pelvicalyceal system (Supplementary Fig. 1).

#### Biochemical assays of Pre-PGS-PFP & Sonovue microbubble-injected dogs

**Figure 8:**
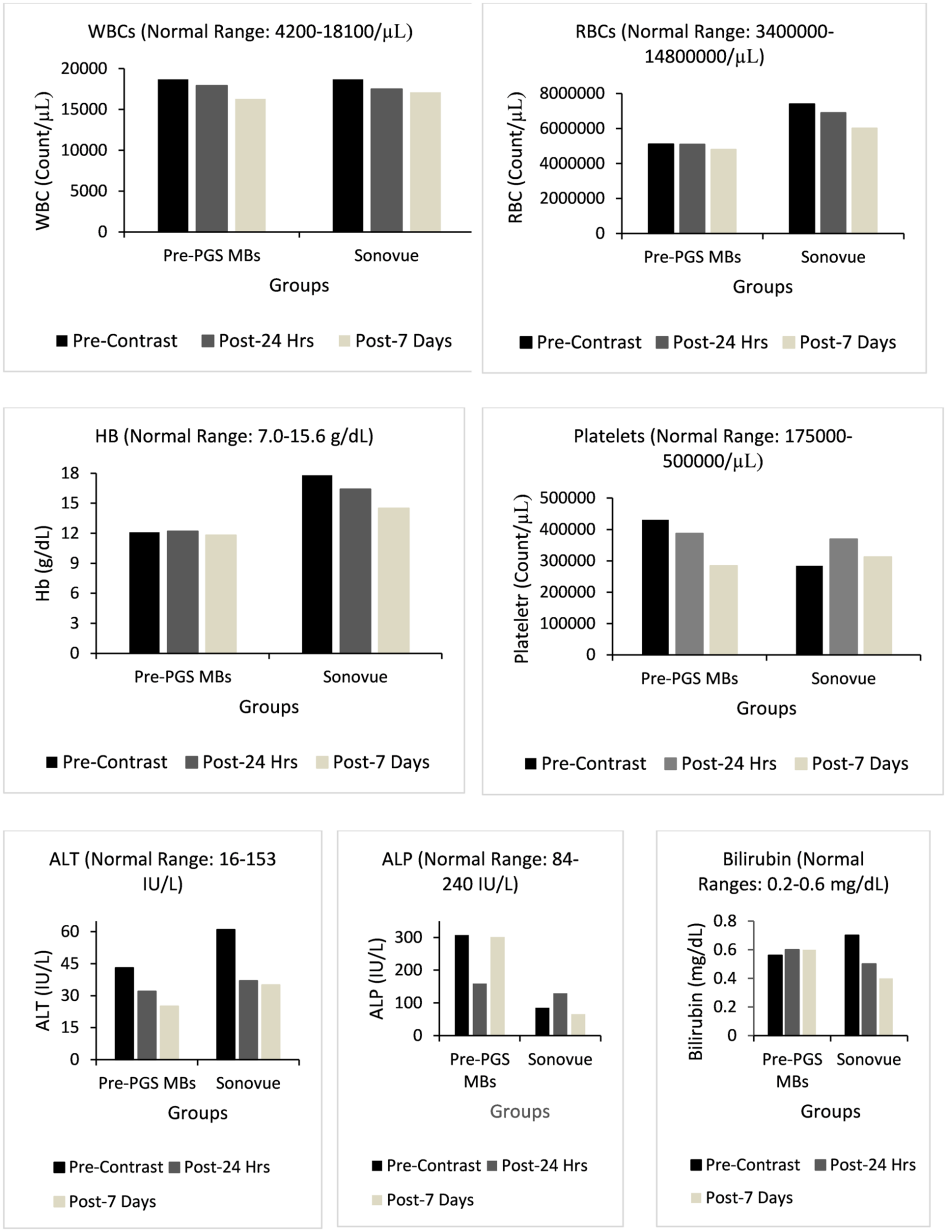
Biochemical assays of pre-PGS & Sonovue MBs administered dogs showing all assays within the normal range before and after contrast administration.

The complete blood count of both microbubble-injected dogs remained almost the same before and after contrast injection. Liver function tests of both dogs also showed similar values for ALT and bilirubin; however, the ALP amount of pre-PGS-PFP-injected dogs was found to be decreased on the 24th hr. reading, which increased again on the 7th day of reading (Fig. 8). Since the decrease in ALP values in the immediate postinjection group was the same in rats and dogs, we searched for the possible reason. We found that ALP has an autofeedback inhibition mechanism depending on phosphate levels in the blood^43^. Since our construct has glycerol, which increases serum phosphate levels, it could be the possible cause of low ALP^44^. In addition, glycerol itself has also been found to inhibit ALP activity and has been used for the protection of serum samples to inhibit extra ALP activity^45^. Oral intake of glycerol monolaurate has also been found to be negatively linked with ALP levels in laying hens^46^. Therefore, circulating glycerol could be the possible reason behind low ALP levels, which is one of the breakdown products of these nanodroplets.

Further renal function tests of both dogs also showed similar results for blood urea and creatinine levels before and after contrast injection, which shows the safety of these nanodroplets. The mild temporary rise in urea levels in both rats and dogs after pre-PGS MB administration could also be attributed to glycerol levels in the blood. Treatment with glycerol was found to be linked with a mild rise in serum urea levels^44^. Figure 8 shows biochemical assays of pre-PGS and Sonovue MB-administered dogs.

## Materials and methods

### Materials

Glycerol (99% purity), sebacic acid (99% purity), and ethanol (99.9% purity) were purchased from Sigma‒Aldrich. PBS tablets were purchased from Oxoid, UK. Ketamine-midazolam was a gift from the hospital solely for research purposes. Aquaflex Ultrasound gel pads were purchased from Parker Laboratories. Hair removal cream was purchased from the commercial market.

### Methods

#### Synthesis of pre-PGS-PFP nanodroplets

For the synthesis of pre-PGS-PFP nanodroplets, pre-PGS was synthesized via a melt condensation reaction adapted from^47^. Briefly, an equimolar ration (1:1) of sebacic acid and glycerol was taken in a three-neck flask and heated for 15 min to ensure homogeneity. Afterward, reaction proceeded at 180 ℃ under continuous nitrogen supply with constant stirring for 3 h. Upon completion of the reaction, yellowish sticky viscous pre-PGS was obtained which changed into a white solid upon cooling. The Pre-polymer was stored at −20 ℃ until further use. Detailed characterization of pre-polymer was performed and published in our previous study^15^. Nanodroplets were then prepared using pre-PGS via the solvent-displacement method. Briefly, a 5% PFP emulsion was prepared in PBS with Tween 80 as a surfactant under probe sonication at 60% amplitude i.e. 120 W via Hielscher Ultrasonicator (UP400S) with a 1 min cycle of sonication preceded by a1 min break upon ice bath for 10 min. The organic phase consisted of a 100% w/v solution of pre-PGS in ethanol with Span 20 as a surfactant. The aqueous phase (20 mL) was extruded via 0.4 um filter and dropwise added into the organic phase (4 mL) under continuous stirring. Solvent evaporation was performed at room temperature with stirring for 3 h. The step was followed by centrifugation at 1000 rpm for 10 min three times. The obtained nanodroplets were lyophilized and stored at −20 ℃. During freeze drying 0.1 mBar pressure was used with 6 h cycle in the lyophilizer followed by 18 h storage at −20 ℃ for 4 days making total lyophilization duration of 24 h inside machine. Because of the viscous nature of polymer, the obtained product was not in powdered form. Rather a concentrated, easy to disperse pellet was obtained at end of lyophilization. This also shows that product remained in its original form and only water was removed during lyophilization. Since shell was made up of polyglycerol sebacate, we did not added any cryoprotectant as glycerol in the shell itself plays a role of cryoprotectant. For morphological analysis of the core and shell, fluorescent nanodroplets with a core labelled with fluorescein and a shell labelled with methyl violet dye were also synthesized. Fluorescein was added in the emulsion formation step, while methyl violet was dissolved with pre-PGS in ethanol. The rest of the methodology remained the same. The synthesized nanodroplets were converted into microbubbles by adding heated PBS and used immediately. To achieve maximum conversion and match it with body conditions, PBS was heated at 37 ℃.

#### Physicochemical characterization of pre-PGS-PFP nanodroplets

For the chemical characterization of nanodroplets, Fourier transform infrared spectroscopy (FTIR) was performed by the KBr disc method using a Perkin Elmer instrument (USA) at wavelengths of 400 – 4000 cm^-1^. For morphological characterization, optical microscopy of both nonconverted nanodroplets and converted microbubbles was performed. To check the core–shell morphology, fluorescent microbubbles were visualized under fluorescent and confocal microscopes. For nonconverted nanodroplets, scanning electron microscopy (SEM) was performed by placing a drop of nanodroplets on a slide followed by a coating of gold and visualization under an SEM-JEOL (JSM-6490 L, Tokyo, Japan) microscope. For phase-converted microbubbles, field-emission SEM was performed by a Nova Nano-SEM 450 scanning electron microscope (FE-SEM, FEI Company, USA). To measure the surface charge and hydrodynamic diameter of the nanodroplets, zeta charge and size analyses were performed using a Malvern Zeta Sizer ver 7.12 **(**Worcestershire**,** UK) at room temperature.

#### In vitro ultrasound imaging

To check the contrast enhancement potential of phase converted pre-PGS microbubbles, in vitro ultrasound imaging using a phased array probe was performed with a central frequency of 3.2 MHz (2–5 MHz). For this, converted microbubbles were counted via a haemocytometer under optical microscopy. Afterward, 1*10^6^ MBs were added to 1000 mL of water. Images were compared with an equal amount of Sonovue MBs. The duration of enhancement was checked at 0.6 MI. Image intensity was calculated using ImageJ software.

#### In vivo contrast efficacy and safety establishment

After obtaining ethical approval from the *Ethical Review Committee, NUST* (IRB no: 02-2021-03-34), in vivo imaging and safety establishment was performed in 20 male Wistar rats in accordance with *ARRIVE 10 Guidelines*. All experiments were performed in accordance with relevant guidelines and regulations. For best practice of anaesthesia and euthanasia in animals, American Veterinary Medical Association *(AVMA) Guidelines* (2020) were followed. A case‒control study design was used. The negative control group was not given any contrast injection, and their biochemical and histological profiles were considered baseline. The saline group was considered a positive control for the contrast effect. The test group was further divided into two groups for the establishment of short- and long-term safety. The study was performed on a small sample size, as the pre-PGS-based droplets were first employed in an animal model. Rats weighing 150–400 kg were included in the study. No rat below 150 kg was included in the study to ensure a good acoustic window during imaging. All animals were housed under similar conditions and kept on a similar diet. No more than 3 rats were housed in one cage. To ensure blinding during result analysis, samples were sent for analysis with coded numbers to the laboratory.

The primary outcome of the study was to establish ventricle opacification by contrast agent, and the other was to establish its safety via biochemical and histopathological analysis. Contrast enhancement was further verified in two dogs. For verification in dogs, standard Sonovue MBs were used as positive contrast controls. The detailed methodology of the in vivo experiments is attached in the supplementary file.

*Statistical analysis* was performed via GraphPad Prism 9. Descriptive statistics included the mean and standard deviation. For inferential analysis, ANOVA was applied. One-way ANOVA was performed for in vivo echocardiography and safety analysis tests. For in vivo echocardiography image analysis, Bonferroni post hoc analysis was performed; for safety assays, Dunnett’s post hoc analysis was performed. A P value < 0.05 was considered significant.

### Institutional review board statement

The study was approved by the institutional review board named as Ethical Review Committee-NUST under IRB no 04-2021-02/34.

## Conclusion

Pre-PGS-PFP MBs showed excellent contrast enhancement in both rats and dogs without any vital organ toxicity or side effects. Contrast enhancement and duration of enhancement were both better for pre-PGS-PFP MBs than Sonovue MBs. This shows its excellent potential to be used for both human and veterinary purposes.

### Supplementary Information

Supplementary Information.

## Data Availability

The datasets used and/or analysed during the current study available from the corresponding author on reasonable request.
