# Supplementary material for: Contrast efficacy of novel phase convertible nanodroplets for safe CEUS imaging
Source: Sci Rep. 2024 Jul 12;14:16126. doi: 10.1038/s41598-024-66163-1 (PMC11245480; doi:10.1038/s41598-024-66163-1)
Supplement: Supplementary file 1 — Supplementary Information. [file 41598_2024_66163_MOESM1_ESM.docx]

**Supplementary Data:**

**S1. Detailed Methodology**

**In vivo Contrast Efficacy & Safety in Rats:**

Rats were divided into 4 groups as explained in schematics:


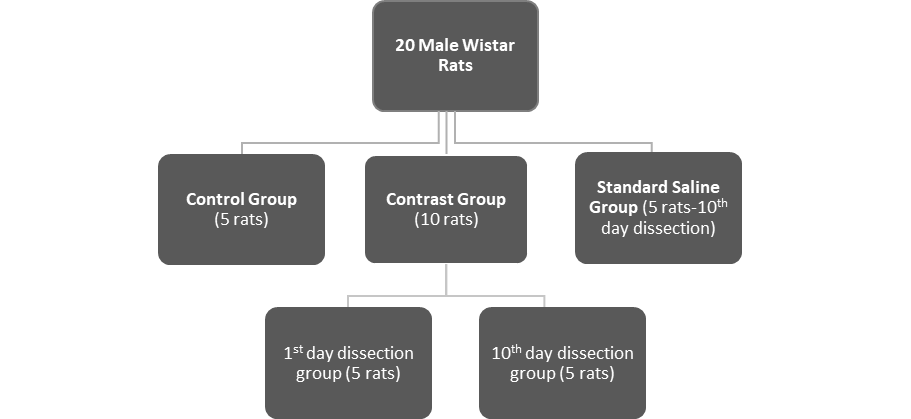


***Echocardiography of rats:***

For imaging purposes, rats were first anesthetized using Ketamine/Midazolam combination with a standard dose of ketamine: 150 to 200 mg/ kg and for midazolam: 5-10 mg/kg diluted in 9 % normal saline. Under anaesthesia, rats were shaved over the chest area after applying hair removing cream. After shaving, pre-contrast echocardiography was performed via GE Logic Book XP having a phased array probe of central frequency 3.2 MHz at 0.6 MI. B-mode and M-mode images were taken. For contrast-enhanced imaging, 7*10^6 microbubbles /300 ul of PBS was injected via rat tail vein. For the agitated saline group, agitated saline was freshly prepared via mixing 0.9 % saline and air using two 10 mL syringes connected through a three-way stop cork and injected rapidly. Post-contrast images were taken at a similar position, image intensities of pre-contrast and post-contrast images were calculated through image J and compared via ANOVA analysis.

***In vivo safety establishment:***

For establishing the safety of nanodroplets, all rats were sacrificed on the 10th day except for one contrast group rats to check the immediate effect, they were dissected after one hour following imaging. Following tests were performed:

*Monitoring Vitals:*

Vitals monitoring was performed for every rat group. The weight of all the rats was noted on the 1st and 10th days. Heart rate, respiratory rate, and temperature were monitored for all the rats before and after administration of the contrast medium. Rats were also observed for any unusual behaviour like change in dietary pattern, sleep pattern, any skin change or secretions from ear, eyes, and nose.

*Biochemical Tests:*

For biochemical analysis, blood was collected via cardiac puncture. Biochemical tests included complete blood picture (CBC), liver function tests (ALTs, ALP, and Bilirubin), and renal function tests (Urea and Creatinine). Comparative analysis was performed via ANOVA analysis.

*Histopathological Analysis:*

Histopathology of six major organs was performed, these included liver, kidney, brain, heart, lungs, and spleen. All organs were washed with 0.9 % saline and weighed before slide preparation. H&E staining was used. Slides were observed under microscope at 4 X and 40 X resolution.

#### Verification of Contrast Efficacy & Safety in dogs:

Successful experiments on rats gave us confidence to check contrast enhancement and safety in a large animal. Therefore, we conducted our trial on dogs however due to limited availability we choose two dogs, in one we injected our pre-PGS-PFP MBs construct while in the other, we gave a standard commercially available Sonovue® contrast agent.

*Echocardiography/Ultrasound of dogs:*

For ultrasound imaging, dogs were given a standard dose of ketamine/xylazine in 1:1 to get adequate anaesthesia. Firstly, pre-contrast echocardiography was performed followed by contrast injection (2.5 *10^8MBs) and post-contrast echocardiography. Since pre-PGS-PFP nanodroplets gave imaging window up to 10 minutes, we utilized this time and also performed ultrasonography of liver and kidney.

*Safety Assays of dogs:*

To check any possible effect, we performed pre-contrast, 24 hr post-contrast injection, and 7 days post-contrast injection CBC, LFTs, and RFTs of both dogs.

**S2. Organ Weight Analysis:**

Before histopathological analysis, every organ was weighed and compared. No significant difference was found between the organ weights of the different groups (Table 1).

Supplementary Table 1 Statistical analysis of the weight of vital organs in the test versus control groups. The P value was considered significant at P< 0.05. ns represents nonsignificant results.

| **Organ** | **Groups** | **Mean Weight ± SD** | **ANOVA** | **P Value** |
| --- | --- | --- | --- | --- |
| **Liver** | Control | 6.86±1.99 | 1.22 | ns |
|  | Contrast (1 day) | 5.70±1.6 |  |  |
|  | Contrast (10 day) | 6.68±1.59 |  |  |
|  | Saline | 4.77±0.77 |  |  |
| Kidney | Control | 0.77±0.13 | 2.05 | ns |
|  | Contrast (1 day) | 0.6±0.03 |  |  |
|  | Contrast (10 day) | 0.71±0.06 |  |  |
|  | Saline | 0.63±0.02 |  |  |
| **Heart** | Control | 0.90±0.21 | 0.74 | ns |
|  | Contrast (1 day) | 0.65±0.48 |  |  |
|  | Contrast (10 day) | 0.76±0.18 |  |  |
|  | Saline | 0.67±0.15 |  |  |
| **Brain** | Control | 1.09±0.24 | 0.80 | ns |
|  | Contrast (1 day) | 0.81±0.73 |  |  |
|  | Contrast (10 day) | 1.20±0.56 |  |  |
|  | Saline | 1.23±0.05 |  |  |
| **Lung** | Control | 1.37±0.32 | 0.52 | ns |
|  | Contrast (1 day) | 1.14±0.03 |  |  |
|  | Contrast (10 day) | 1.14±0.16 |  |  |
|  | Saline | 1.07±0.11 |  |  |
| **Spleen** | Control | 0.32±0.07 | 2.0 | ns |
|  | Contrast (1 day) | 0.22±0.03 |  |  |
|  | Contrast (10 day) | 0.28±0.08 |  |  |
|  | Saline | 0.35±0.01 |  |  |

**S3- Abdominal Ultrasound Imaging:**

Pre-PGS-PFP MBs gave a longer imaging window. This provided us with an opportunity to image the liver and kidney of the dog after five to six minutes of echocardiography. The liver retained contrast and showed slow washout. However, when the kidney was observed after the liver, very little contrast was present in the pelvicalyceal system. Supplementary Figure 1 shows these findings.

IVS

| 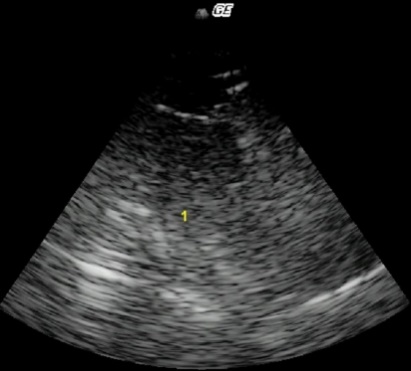  (a) | 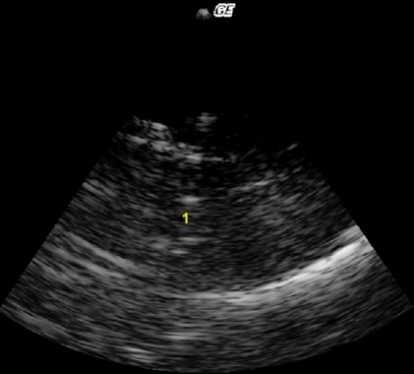  (b) | 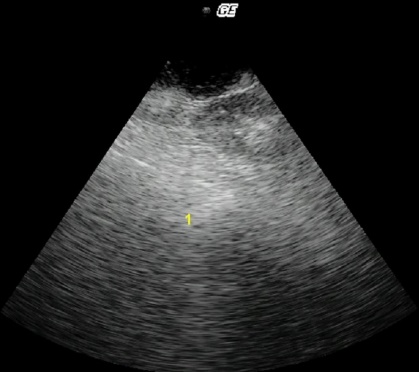  (c) |
| --- | --- | --- |

Supplementary Figure 1 Liver showing contrast enhancement (a), contrast washout (b) and kidney showing minimal contrast in the pelvicalyceal system (c).
